# The correlation and role analysis of SLC30A1 and SLC30A10 in cervical carcinoma

**DOI:** 10.7150/jca.56777

**Published:** 2022-01-04

**Authors:** Jing Zhang, Xin-Wei Chen, Li-Sha Shu, Chong-Dong Liu

**Affiliations:** 1 Department of gynecology and obstetrics, Chao Yang Hospital of Capital Medical University, Beijing,100020, China; 2 Department of blood transfusion, The First Affiliated Hospital of Hebei North University, Zhangjiakou, 075000, China; 3 Department of gynecology and obstetrics, The First Affiliated Hospital of Hebei North University, Zhangjiakou, 075000, China

**Keywords:** cervical carcinoma, SLC30A1, SLC30A10, microenvironment

## Abstract

**Background**: SLC30 family genes, also known as ZnT family genes, can keep cellular zinc levels within a physiological range by exporting zinc to extracellular space or by isolating zinc in the specific regions of cytoplasm when cellular zinc concentrations are elevated in human cells. There are growing evidences that dysregulated expression of SLC30 family genes can potentially influence tumorigenesis. However, the expression and prognostic value of SLC30 family genes in cervical carcinoma are poorly characterized.

**Methods**: In this study, we used many tools such as UALCAN, Kaplan-Meier Plotter, cBioPortal, LinkedOmics, FunRich, Metascape, GeneMANIA, Open targets and TISIDB to perform bioinformatics analysis of SLC30 family genes in cervical carcinoma.

**Results:** We found that the expression of SLC30A1/7/10 was significantly higher in cervical carcinoma than that in normal matched tissues, while SLC30A2/8 mRNA levels were decreased compared to normal tissues. For tumor stages, SLC30A1, SLC30A7 and SLC30A10 groups significantly varied. And a high expression of SLC30A1, SLC30A6, SLC30A8 and SLC30A10 was associated with worse overall survival in cervical carcinoma patients. Besides, we found that SLC30A1/10 may have a potential regulatory role in immune infiltration in cervical carcinoma. In addition, the results showed that the high expression of SLC30A1 was resistant to 79 drugs or small molecules; Two drugs (Neopeltolide and Tozasertib) can inhibit the high expression of SLC30A10 in cancers.

**Conclusion**: SLC30A1 and SLC30A10 can be recognized as potential diagnostic indicators and therapeutic targets in cervical carcinoma.

## Introduction

As one of the common malignant neoplasms of female reproductive system, the incidence of cervical malignancy is increasing. By the end of 2018, there were a total of 569,847 new cases of cervical cancer worldwide, of which 311,365 cases died from cancers or cancer-related metastatic diseases 1. In addition, compared with other cancers, cervical cancer has become the top-one reproductive malignancy among Chinese women, and the mortality rate of cervical cancer is gradually increasing in China 2. Although the incidence rate of cervical cancer has remained relatively stable in developed countries, the prevalence of cervical cancer in developing countries remains high. In particular, lymph node metastases can occur in the early stage of cervical cancer, which directly reduces the efficacy of treatment 3, 4. For example, the 5-year survival rate for cervical cancer patients with intrapelvic lymph node metastases is 50%-60%, while the 5-year survival rate for patients with metastases in the para-aortic lymph nodes is only 20%. And for the patients diagnosed with advanced cervical cancer, the 5-year survival rate was about 5%-15% 5. Therefore, the exploration of the new molecular target in cervical cancer is important to investigate the potential mechanism of cervical carcinogenesis.

It has been reported that zinc (Zn^2+^) can be involved in the regulation of cancer immune regulation and growing evidence has suggested that zinc is strongly associated with tumor development and metastasis 6. SLC30 family genes, also known as ZnT family genes, can keep cellular zinc levels within a physiological range by exporting zinc to the extracellular space or by isolating zinc in specific regions when cellular zinc concentrations are elevated 7. Currently, ten SLC30 family member genes are known, from SLC30A1 through SLC30A10. It has been proposed that SLC30 family genes can be involved in regulating the malignant biological behavior in a variety of cancer cells. For example, Guo et al noted that SLC30A5 and SLC30A7 were highly expressed in gastric cancer compared to paracancerous tissues and SLC30A5-7 can be used as a diagnostic and prognostic indicator for gastric cancer 8. In addition, upregulation of SLC30A1 expression in ovarian cancer cells counteracted the apoptotic effects of microRNA-8073 mimics on SKOV3 and OVCAR3 cells, indicating that SLC30A1 has anti-apoptotic effects 9. Besides, Bostanci et al stated that inhibiting the expression of SLC30A2 expression could decrease the invasive capacity of MDA-MB-453 breast cancer cells by downregulating the matrix metalloproteinase 2 (MMP-2) expression 10. And SLC30A4 was overexpressed in prostate cancer compared to paracancerous tissues, and SLC30A4 was negatively correlated with the intensity of immunoreactivity in prostate cancer 11. In this study, we performed integrated bioinformatics analyses of SLC30A family genes in cervical cancer.

## Materials and Methods

### Tissue samples

The cervical cancer tissue microarray was bought from Shanghai Outdo Biotech Co., Ltd. The tissue microarray was consisted of 31 cervical squamous cell carcinoma (mean age 41.0±2.6) tissues and paired adjacent non-tumor tissues. Patients meeting the following criteria were included: (1) all patient samples in this tissue microarray were pathologically diagnosed as cervical squamous cell carcinoma; (2) all patient samples required no additional treatment. And the exclusion criteria were as follows: patients with a history of chemotherapy or radiotherapy. The detailed clinic parameters of the enrolled patients were illustrated in Table 1. The present study was approved by the Research Ethics Committee of Chao Yang Hospital of Capital Medical University. All patients provided informed consent for use of their samples.

### UALCAN analysis

In this study, we first compared the mRNA levels of SLC30 family genes in cervical cancer samples with those in normal tissues.Then the expression of SLC30 family genes at different clinical stages and in different metastatic conditions was analyzed using UALCAN tool 12.

### TCGA data and cBioPortal analysis

The mRNA expression data of SLC30 family genes in 275 cervical carcinoma samples were acquired from the Cancer Genome Atlas (TCGA) database 13. The cBioPortal dataset for cervical squamous cell carcinoma and endocervical adenocarcinoma (TCGA, PanCancer Atlas) (including data from 275 pathology reports) was then analyzed for further analysis.

### The Kaplan-Meier plotter analysis

To assess the prognostic value of the SLC30 family genes in cervical cancer, survival analysis was performed online using the Kaplan-Meier website to obtain overall survival results 14. A log P-value <0.05 was set as statistically significant.

### LinkedOmics analysis

The genes positively or negatively correlated with SLC30A1 and SLC30A10 in cervical carcinoma were visualized by using the LinkFinder module 15. The kinase-target, miRNA-target and transcription factor-target analysis of SLC30A1 and SLC30A10 were analyzed using the LinkInterpreter module.

### Functional enrichment analysis

FunRich (Functional Enrichment analysis tool) was a software program for functional enrichment and interaction network analysis of certain genes 16. Then, the FunRich software was used to perform a functional enrichment analysis of the SLC30 family genes. Metascape was a platform for network construction and functional analysis of proteins by integrating multiple authoritative database resources 17. Likewise, we used the Metascape platform to perform a functional enrichment analysis of SLC30 family genes with their closely related genes. Finally, to clarify whether the SLC30A1-10 genes were involved in the regulation of cancer pathways, we performed a correlation analysis using the GSCALite tool 18.

### STRING and GeneMANIA analysis

In this experiment, we first analyzed possible connections between SLC30 family genes using the STRING tool 19. Besides, we constructed a protein-protein interaction network using the GeneMANIA website for SLC30 family genes as well as genes closely related to their functions 20.

### Open targets analysis

Open Targets was a platform for predicting diseases associated with specific genes 21. Here, Open targets was used to explore diseases related to SLC30A1 gene and SLC30A10 gene, respectively.

### Gene set enrichment analysis

GSEA (Gene Set Enrichment Analysis) used a predefined set of genes to sort genes according to their differential expression between two types of samples, and then it checked whether the predefined set of genes was enriched at the top or bottom of the set. Here, the potential roles of SLC30A1 and SLC30A10 genes in the development of cervical cancer were investiged by GSEA. Data were considered significant at *P*<0.05.

### TISIDB analysis

The TISIDB tool is an online tool that can be used to analyze the immune-related effects of specific genes 22. In this experiment, we evaluated the possible regulatory factors and immunological subtypes involved in the immunoregulation of SLC30A1 and SLC30A10 in cervical cancer.

### Immunohistochemical (IHC) staining and evaluation

Immunostaining of SLC30A1 and SLC30A10 was conducted using a rabbit polyclonal anti-SLC30A1 antibody (1:500, Cat. ab246910, Abcam), a rabbit polyclonal anti-SLC30A10 antibody (1:200, Cat. ab229954, Abcam). Overall IHC score of 1-5 as assessed by Remmele's semi-quantitative immune response score (IRS) scale 23. Assessment of IHC staining was independently performed by two expert pathologists. SLC30A1 and SLC30A10 protein expressions were scored according to staining intensity and the percentage of positive cells. The percentage of positive cells was scored as follows: 0 (0-5%); 1 (6-25%); 2 (26-50%); 3 (51-75%); and 4 (76-100%). Staining intensity was scored as follows: 0 (negative); 1-2 (weak); and >2 (strong). Comprehensive score = staining percentage × intensity. SLC30A1 and SLC30A10 protein expressions were classified as follows: < 5 low expression, ≥ 5 high expression.

### Statistical methods

ROC curves were conducted using the GraphPad Prism 7 software. p <0.05 was considered statistically significant.

## Results

### Relationship between the mRNA levels of SLC30s and the clinicopathological parameters of patients with cervical carcinoma

**Figure 1:**
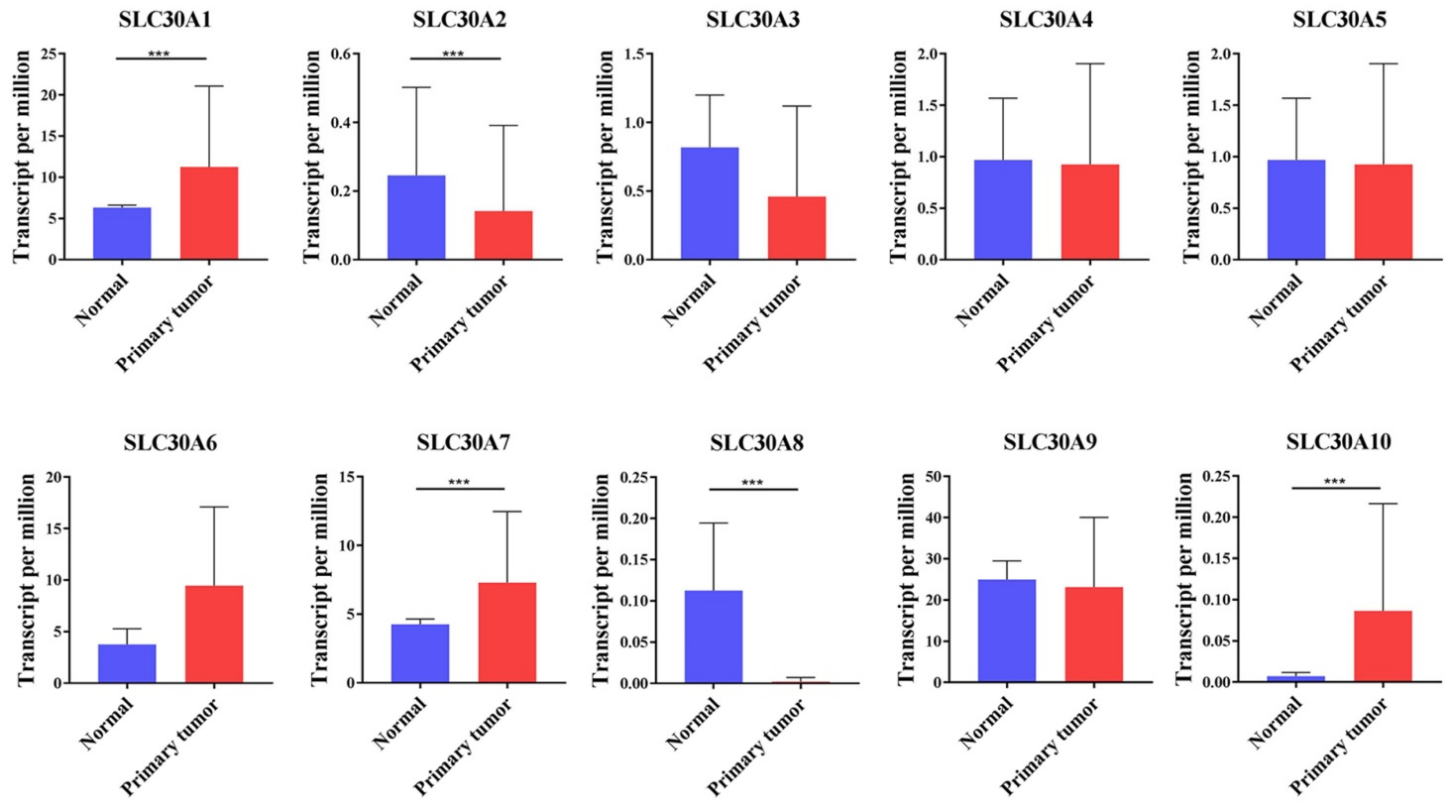
The transcription levels of SLC30A1-10 genes in cervical carcinoma (UALCAN). Compared with normal samples, SLC30A1/7/10 mRNA was overexpressed, and SLC30A2/8 mRNA was underexpressed. **p<0.01, *** p<0.001.

Here, the SLC30 family genes included 10 members, namely SLC30A1, SLC30A2, SLC30A3, SLC30A4, SLC30A5, SLC30A6, SLC30A7, SLC30A8, SLC30A9, SLC30A10. To clarify the expression of these genes, the UALCAN database was employed to analyze the transcript levels of SLC30 family genes in cervical cancer. Data in the UALCAN database revealed that mRNA expression of SLC30A1 (p=1.99651406518342E-12), SLC30A7 (p=3.075600E-03) and SLC30A10 (p=2.64249733206157E-11) were significantly higher in cervical cancer tissues, while SLC30A2 (p=4.340500E-03) and SLC30A8 (p=4.064400E-03) mRNA levels were decreased compared to normal tissues. However, the expression of SLC30A3, SLC30A4, SLC30A5, SLC30A6 and SLC30A9 genes in cervical cancer was not statistically significant compared to the corresponding paracancerous tissues (Figure 1).

**Figure 2:**
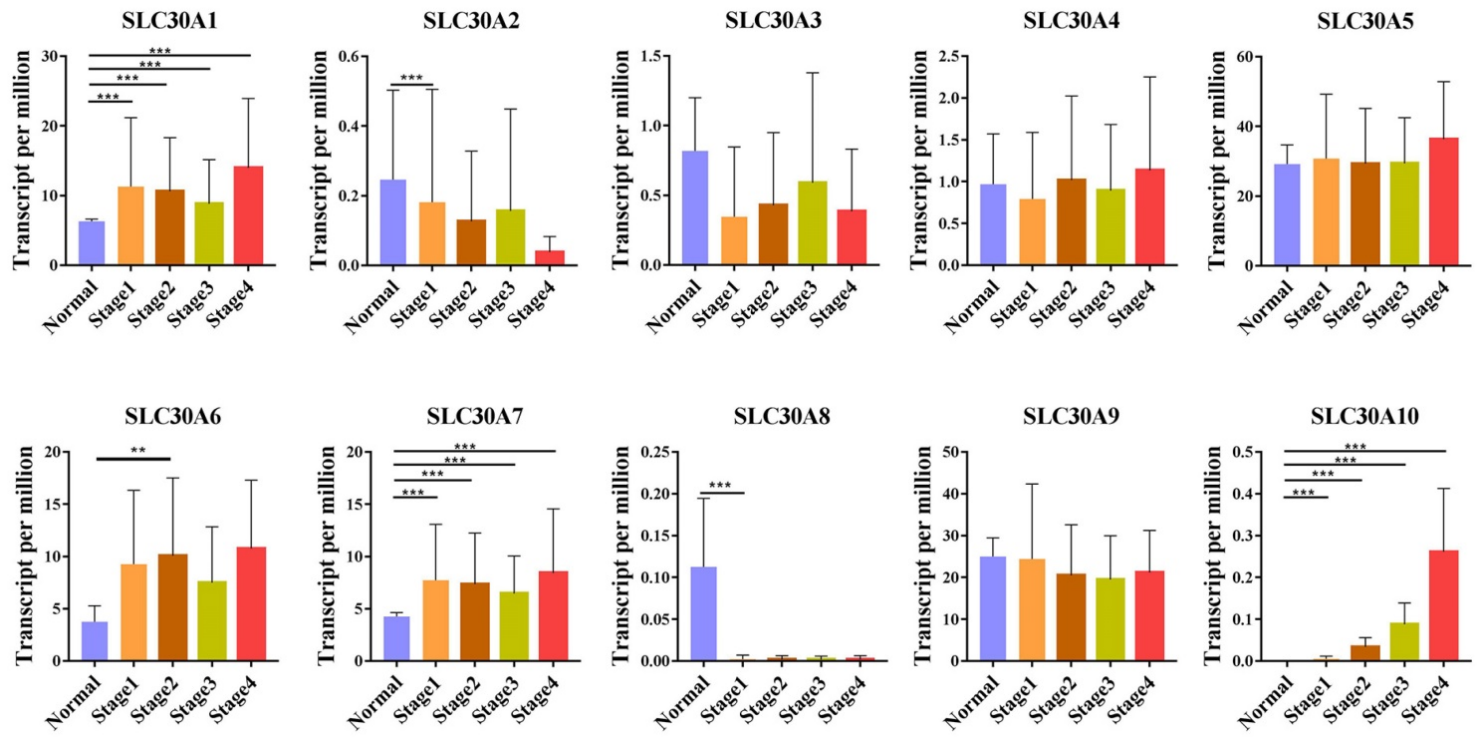
Correlation between SLC30A1-10 genes expression and tumor stage in cervical carcinoma (UALCAN). *p<0.05, **p<0.01, ***p<0.001.

**Figure 3:**
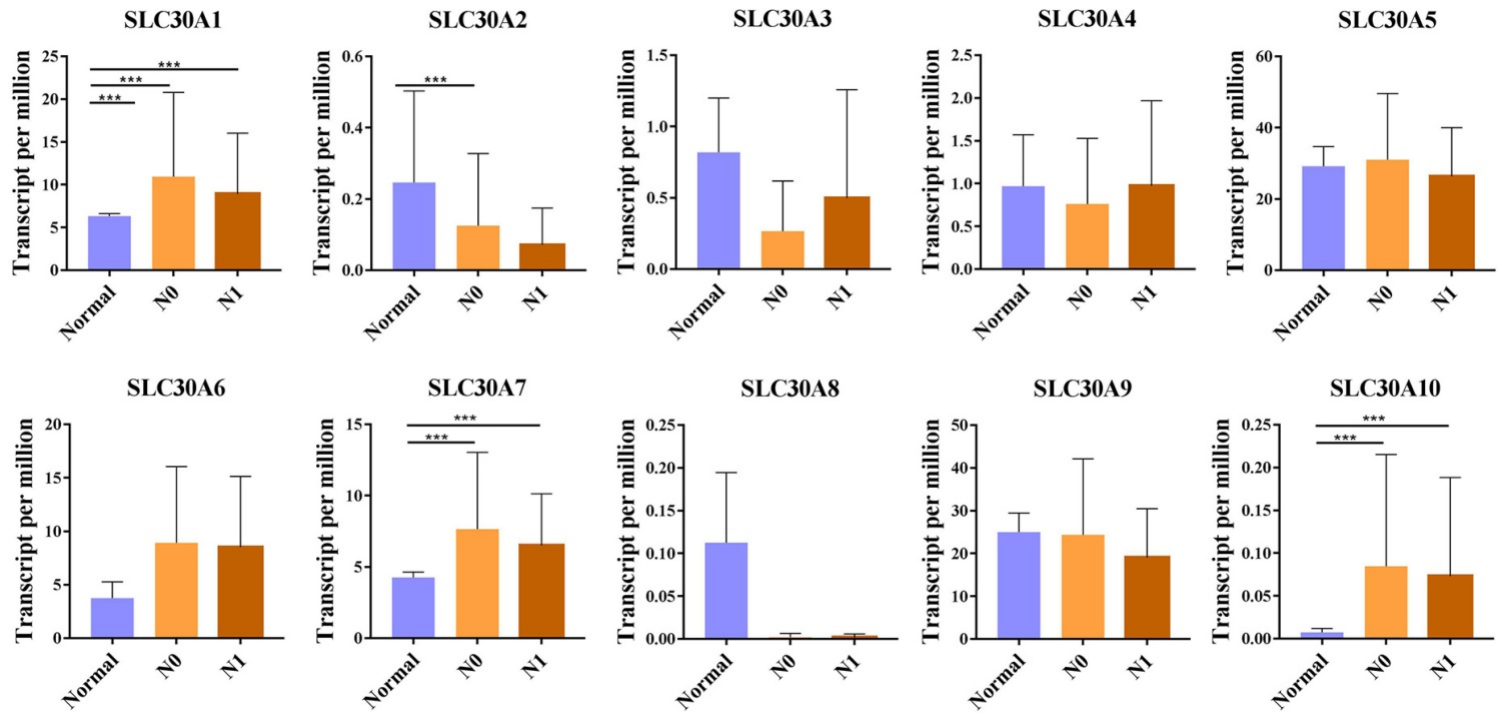
Correlation between SLC30A1-10 genes expression and tumor metastases in cervical carcinoma (UALCAN). *p<0.05, **p<0.01, ***p<0.001.

For tumor stages, SLC30A1, SLC30A7 and SLC30A10 groups significantly varied, whereas SLC30A2, SLC30A3, SLC30A4, SLC30A5, SLC30A6, SLC30A8 and SLC30A9 did not significantly differ in cervical carcinoma (Figure 2). For metastases, we found that SLC30A1, SLC30A7 and SLC30A10 expression were positively correlated with lymph node metastasis or distant metastasis in cervical carcinoma. However, the expression of SLC30A2-6, SLC30A8-9 was not associated with cervical cancer metastasis (Figure 3).

### Functions pathways and altered neighbor genes of SLC30s in cervical carcinoma

**Figure 4:**
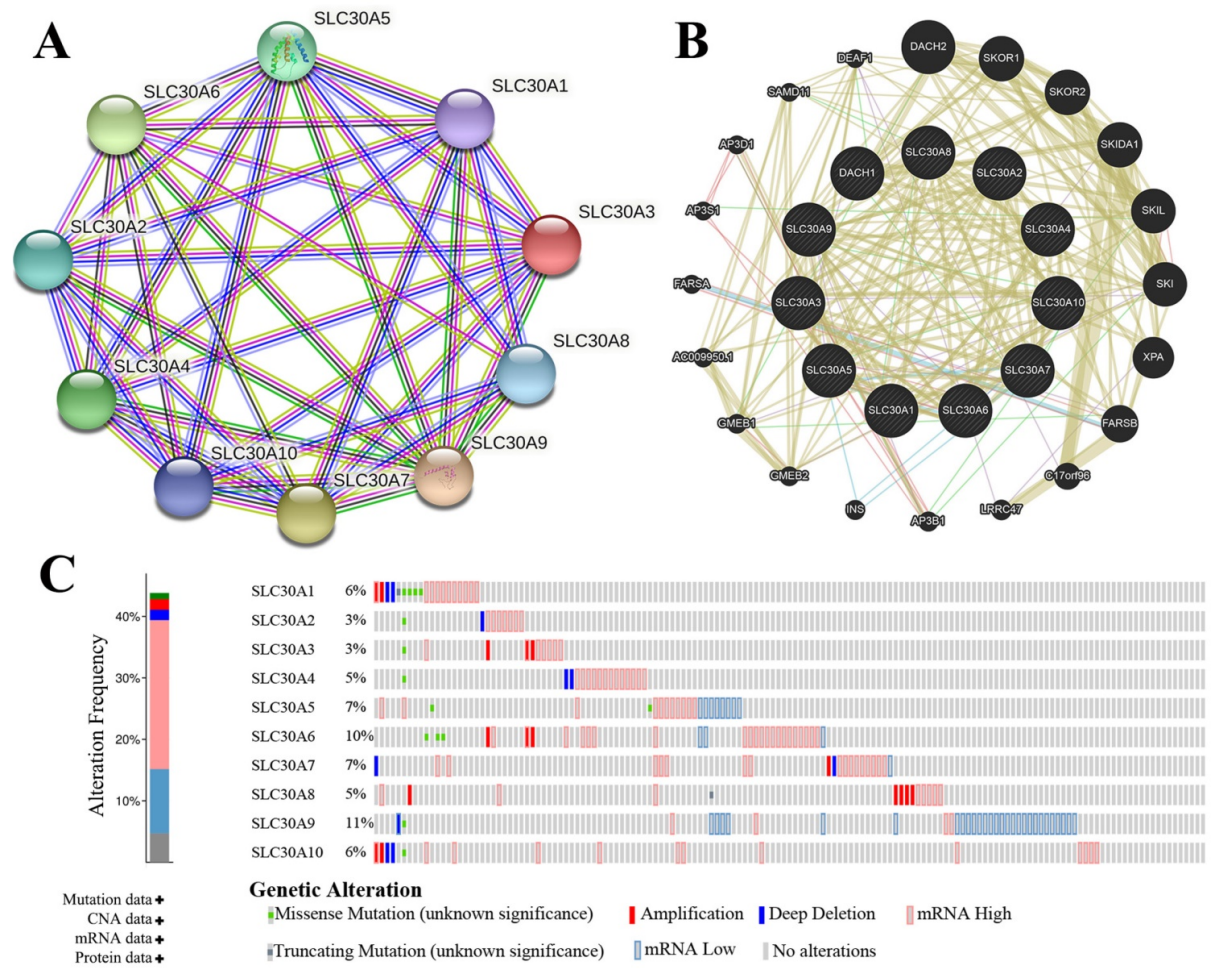
Genetic mutations in SLC30A1-10 genes and their association neighbor genes network (STRING, GeneMANIA and cBioPortal). (A) The network contains 10 nodes. (B) Protein-protein interaction network of SLC30A1-10 genes. (C) Summary of alterations in differently expressed SLC30A1-10 genes in cervical carcinoma.

The genetic alterations of SLC30s in cervical carcinoma were analyzed using cBioPortal (TCGA, PanCancer Atlas). SLC30 family genes were altered in 130 samples of 275 patients with cervical carcinoma (44%). The total overall mutation rate of SLC30s was 43.77% in cervical carcinoma patients, with individual rates for SLC30A1, SLC30A2, SLC30A3, SLC30A4, SLC30A5, SLC30A6, SLC30A7, SLC30A8, SLC30A9, SLC30A10 of 6%, 3%, 3%, 5%, 7%, 10%, 7%, 5%, 11%, 6% (Figure 4C). To elucidate the potential molecular mechanisms of SLC30s in cervical carcinoma, we constructed a regulatory network between SLC30A1-10 using the STRING tool and GeneMANIA tool, respectively (Figure 4A-4B). Besides, the 50 most frequently altered neighboring genes of SLC30 family genes were explored by STRING tool (Figure S1).

### Functional enrichment analysis of SLC30 family genes

**Figure 5:**
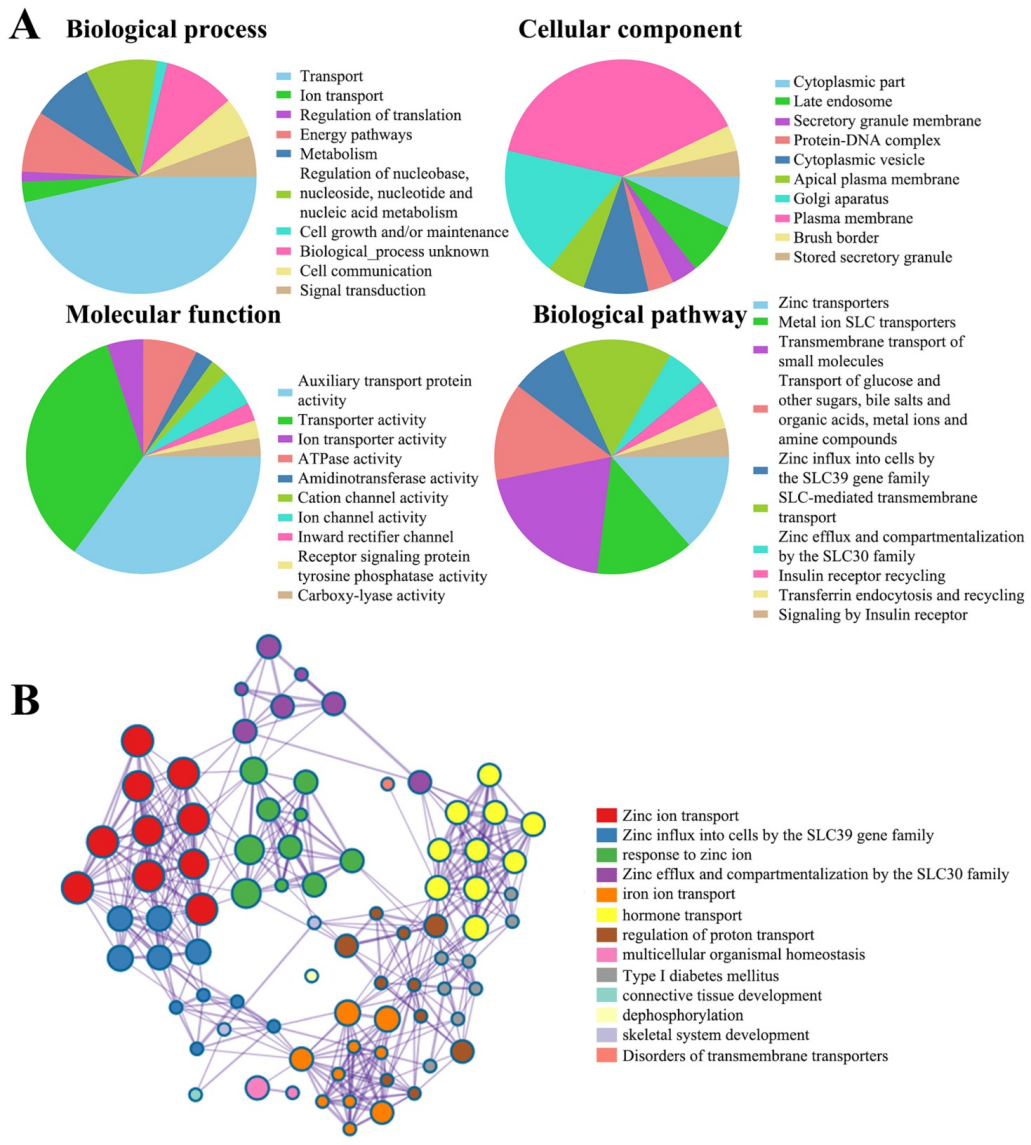
The function of SLC30A1-10 genes and the significant genes associated with SLC30A1-10s changes were analyzed by various analysis tools. (A) Funrich analysis. (B) Metascape analysis.

**Figure 6:**
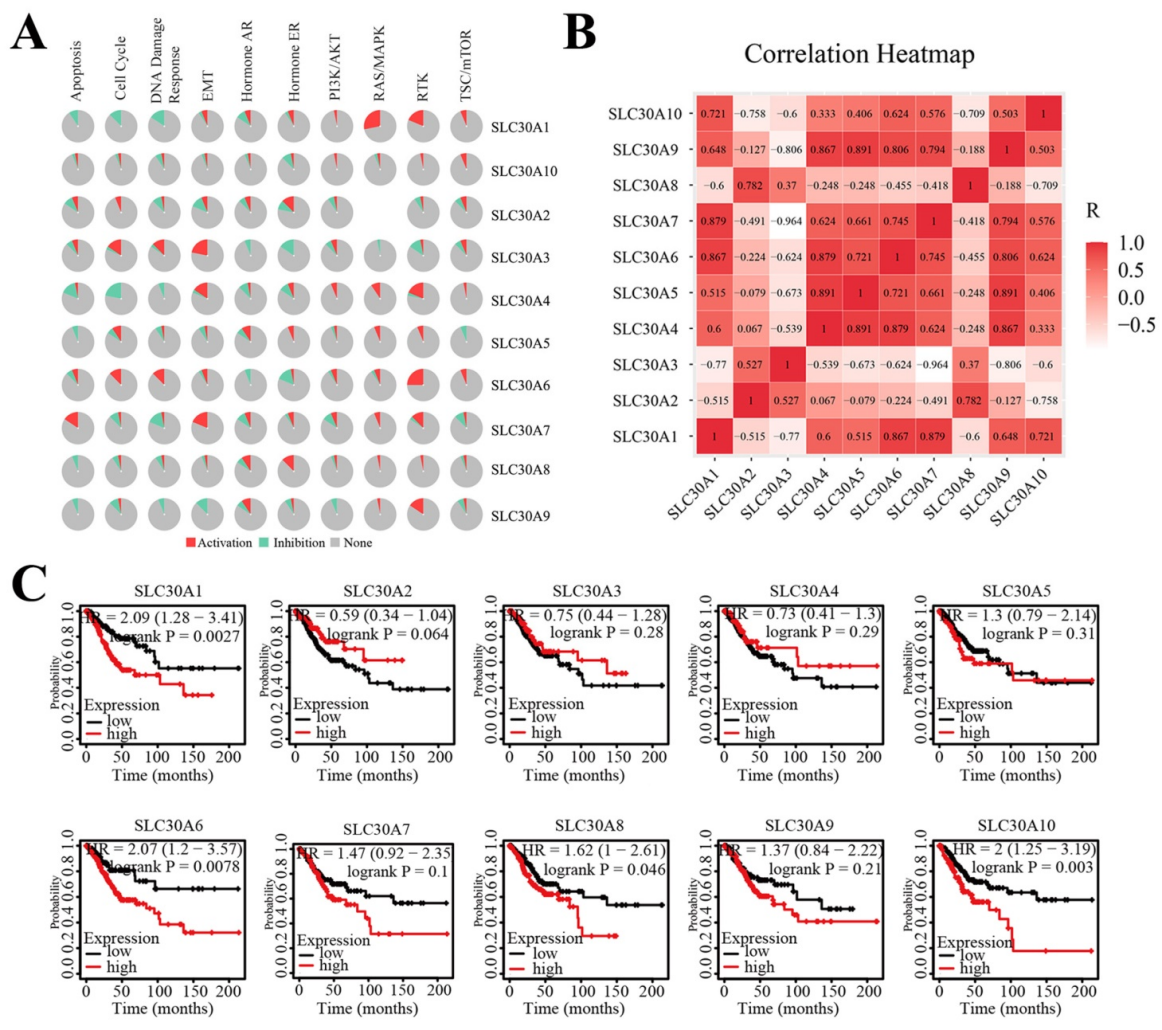
The cancer related pathways (A), correlation heat map (B), survival analysis (C) of SLC30A1-10 genes in cervical carcinoma.

Next, to clarify the underlying mechanisms involved in the regulatory role of SLC30 family genes in cervical cancer, we performed a functional enrichment analysis by using Funrich software and the Metascape platform. The Funrich software predicted the functions of the SLC30 family genes with their closely related TOP-50 genes from four perspectives: biological process, cellular component, molecular function, and related pathways. In the biological process, the roles of these genes were enriched in transport (55%), ion transport (3.33%), regulation of translation (1.6%), energy pathways (10%), metabolism (10%), cell growth (1.6%), cell communication (6.7%), signal transduction (6.7%). Cytoplasmic part (6.6%), late endosome (6.6%), secretory granule membrane (3.3%), protein-DNA complex (3.3%), cytoplasmic vesicle (8.3%), apical plasma membrane (5%), golgi apparatus (16.6%), plasma membrane (36.6%), brush border (3.3%) and stored secretory granule (3.3%) were the ten most highly involved items in the cellular component category. Among the 10 most highly enriched functions in the molecular function category, auxiliary transport protein activity (23.3%), transporter activity (23.3%), ion transporter activity (3.3%), ATPase activity (5.0%), amidinotransferase activity (1.7%), cation channel activity (1.7%), ion channel activity (3.3%), inward rectifier channel (1.7%), receptor signaling protein tyrosine phosphatase activity (1.7%), carboxy-lyase activity (1.7%) were associated with the tumorigenesis of cervical cancer. For biological pathway, the roles of these genes were enriched in zinc transporters (28.3%), metal ion SLC transporters (28.3%), transmembrane transport of small molecules (41.7%), zinc influx into cells by the SLC39 gene family (16.7%), SLC-mediated transmembrane transport (31.7%), zinc efflux and compartmentalization by the SLC30 family (11.7%), insulin receptor recycling (8.3%), transferrin endocytosis and recycling (6.7%) (Figure 5A). Similarly, figure 5B showed the functional enrichment analysis results obtained from Metascape concerning the SLC30 family genes and their closely related genes. The functions of SLC30 family genes and their neighboring genes were mainly concentrated on zinc ion transport, response to zinc ion, iron ion transport, hormone transport, regulation of proton transport, connective tissue development, dephosphorylation, and disorders of transmembrane transporters. To further explore whether the SLC30 family genes could exert oncogenic or tumor-suppressive effects through cancer-related pathways, we used the GSCALite platform to investigate the regulatory effects of the SLC30A1-10 genes on common cancer pathways. As shown in Figure 6A, all SLC30 family genes were engaged in the regulation of cancer-related pathways. In addition, we also explored the correlations between all SLC30s genes. We found that there were asignificant positive correlations between SLC30s family genes (Figure 6B).

### SLC30A1/6/8/10 levels were associated with overall survival of cervical carcinoma

The Kaplan-Meier platform was used to examine the prognostic value of SLC30 family genes in cervical cancer. The results revealed that high expression of SLC30A1 (HR=2.09, 95% CI: 1.28-3.41, and log-rank P=0.0027), SLC30A6 (HR=2.07, 95% CI: 1.2-3.57, and log-rank P=0.0078), SLC30A8 (HR=1.62, 95% CI: 1-2.61, and log-rank P=0.046) and SLC30A10 (HR=2, 95% CI: 1.25-3.19, and log-rank P=0.003) was associated with worse overall outcome in cervical carcinoma patients (Figure 6C).

In conclusion, we identified two genes (SLC30A1 and SLC30A10) that may play a promotional role in cervical cancer. Next, we will specifically explore the potential cancer-promoting mechanisms of these two genes.

### The correlation between SLC30A1/10 expression and immune infiltration in cervical carcinoma

**Figure 7:**
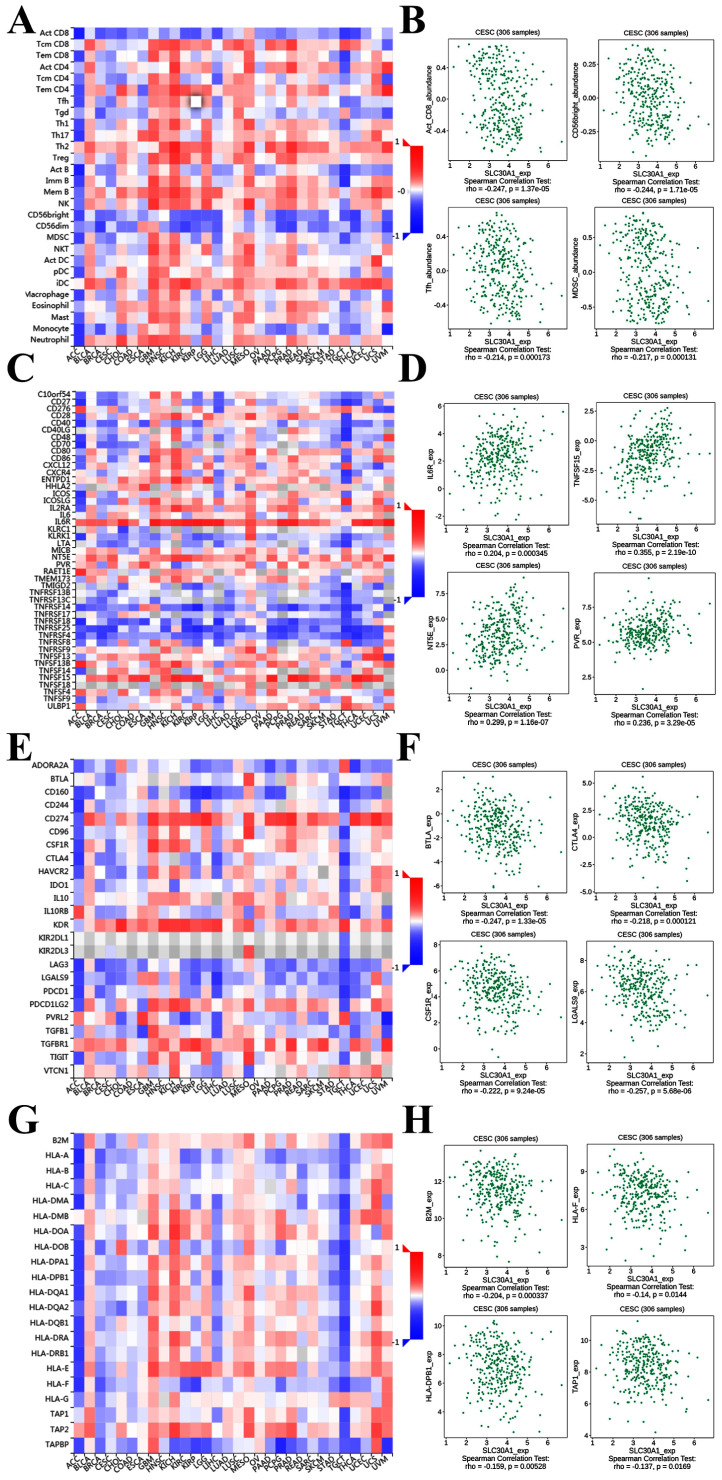
Correlation of SLC30A1 expression with immune cell and immunomodulators in cervical carcinoma. (A) Relations between the tumor-infiltrating lymphocytes and SLC30A1 expression. (B) 4 tumor-infiltrating lymphocytes were correlated with SLC30A1 expression. (C) Relations between the immunostimulators and SLC30A1 expression. (D) 4 immunostimulators were correlated with SLC30A1 expression. (E) Relations between the immunoinhibitors and SLC30A1 expression. (F) 4 immunoinhibitors were correlated with SLC30A1 expression. (G) Relations between the MHC molecules and SLC30A1 expression. (H) 4 MHC molecules were correlated with SLC30A1 expression.

It was well documented that zinc was directly involved in immune regulation and tumor-formation 6. Therefore, the correlation of SLC30A1/10 expression with immune markers was investigated in cervical cancer using the TISIDB platform. Figure 7A demonstrated correlations between SLC30A1 expression and tumor-infiltrating lymphocytes. The tumor-infiltrating lymphocytes showed moderate correlations with SLC30A1 expression including Activated CD8 T cell (Spearman: ρ = -0.247, P = 1.37e-05), CD56 bright natural killer cell (Spearman: ρ = -0.244, P = 1.71e-05), T follicular helper cell (Spearman: ρ = -0.214, P = 0.000173), and Myeloid derived suppressor cell (MDSC) (Spearman: ρ = -0.217, P = 0.000131) in cervical carcinoma (Figure 7A-7B). For immunostimulator, SLC30A1 expression positively correlated with IL6R (Spearman: ρ = 0.204, P = 0.000345), TNFSF15 (Spearman: ρ = 0.355, P = 2.19e-10), NT5E (Spearman: ρ = 0.299, P = 1.16e-07), and PVR (Spearman: ρ = 0.236, P = 3.29e-05) in cervical carcinoma (Figure 7C-7D). Figure 7E-7F revealed correlations between SLC30A1 expression and immunoinhibitor. SLC30A1 expression negatively correlated between and BTLA (Spearman: ρ = -0.247, P = 1.33e-05), CTLA4 (Spearman: ρ = -0.218, P = 0.000121), CSF1R (Spearman: ρ = -0.222, P = 9.24e-05), and LGALS9 (Spearman: ρ = -0.257, P = 5.68e-06). For MHC molecule, SLC30A1 expression negatively correlated with B2M (Spearman: ρ = -0.204, P = 0.000337), HLA-F (Spearman: ρ = -0.14, P = 0.0144), HLA-DPB1 (Spearman: ρ = -0.159, P = 0.00528), and TAP1 (Spearman: ρ = -0.137, P = 0.0169) (Figure 7G-7H).

**Figure 8:**
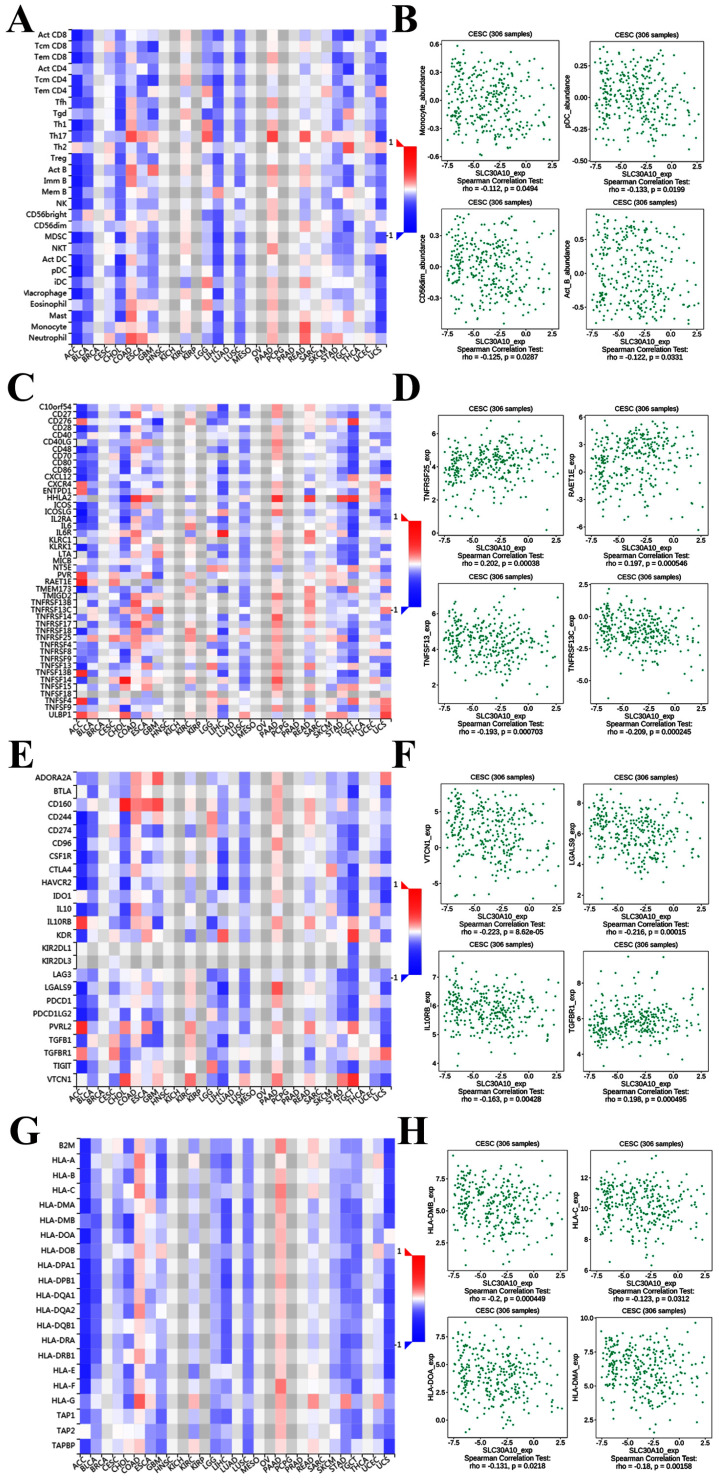
Correlation of SLC30A10 expression with immune cell and immunomodulators in cervical carcinoma. (A) Relations between the tumor-infiltrating lymphocytes and SLC30A10 expression. (B) 4 tumor-infiltrating lymphocytes were correlated with SLC30A10 expression. (C) Relations between the immunostimulators and SLC30A10 expression. (D) 4 immunostimulators were correlated with SLC30A10 expression. (E) Relations between the immunoinhibitors and SLC30A10 expression. (F) 4 immunoinhibitors were correlated with SLC30A10 expression. (G) Relations between the MHC molecules and SLC30A10 expression. (H) 4 MHC molecules were correlated with SLC30A10 expression.

Figure 8A demonstrated correlations between SLC30A10 expression and tumor-infiltrating lymphocytes. The tumor-infiltrating lymphocytes exhibited weak correlations with SLC30A10 expression including Monocyte (Spearman: ρ = -0.112, P = 0.0494), Plasmacytoid dendritic cell (pCD) (Spearman: ρ = -0.133, P = 0.0199), CD56 dim natural killer cell (CD56dim) (Spearman: ρ = -0.125, P = 0.0287) and Activated B cell (Act_B) (Spearman: ρ = -0.122, P = 0.0331) in cervical carcinoma (Figure 8A-8B). For immunostimulator, SLC30A10 expression was correlated with TNFRSF25 (Spearman: ρ = 0.202, P = 0.00038), TNFSF13 (Spearman: ρ = -0.193, P = 0.000703), TNFRSF13C (Spearman: ρ = -0.209, P = 0.000245), and RAET1E (Spearman: ρ = 0.197, P = 0.000546) in cervical carcinoma (Figure 8C-8D). Figure 8E-8F revealed correlations between SLC30A10 expression and Immunoinhibitor. There were negative correlations between SLC30A10 expression and VTCN1 (Spearman: ρ = -0.233, P = 8.62e-05), LGALS9 (Spearman: ρ = -0.216, P = 0.00015), IL10RB (Spearman: ρ = -0.163, P = 0.00428). And SLC30A10 was positively correlated with TGFBR1 (Spearman: ρ = 0.198, P = 0.000495). For MHC molecule, SLC30A10 expression was negative correlated with HLA-DMB (Spearman: ρ = -0.2, P = 0.000449), HLA-C (Spearman: ρ = -0.123, P = 0.0312), HLA-DOA (Spearman: ρ = -0.131, P = 0.0218), and HLA-DMA (Spearman: ρ = -0.18, P = 0.00158) (Figure 8G-8H). Besides, to further demonstrate that whether SLC30A1/10 in cervical carcinoma may have a potential regulatory role in immune infiltration, we analyzed the relationship between SLC30A1/10 and immune infiltrating cells. After using various analytical tools, as shown in Table 2-3, we found that SLC30A and SLC3010 were both negatively correlated with common immune infiltrating cells in cervical cancer.

### Regulators and validation of SLC30A1/10 in cervical carcinoma

**Figure 9:**
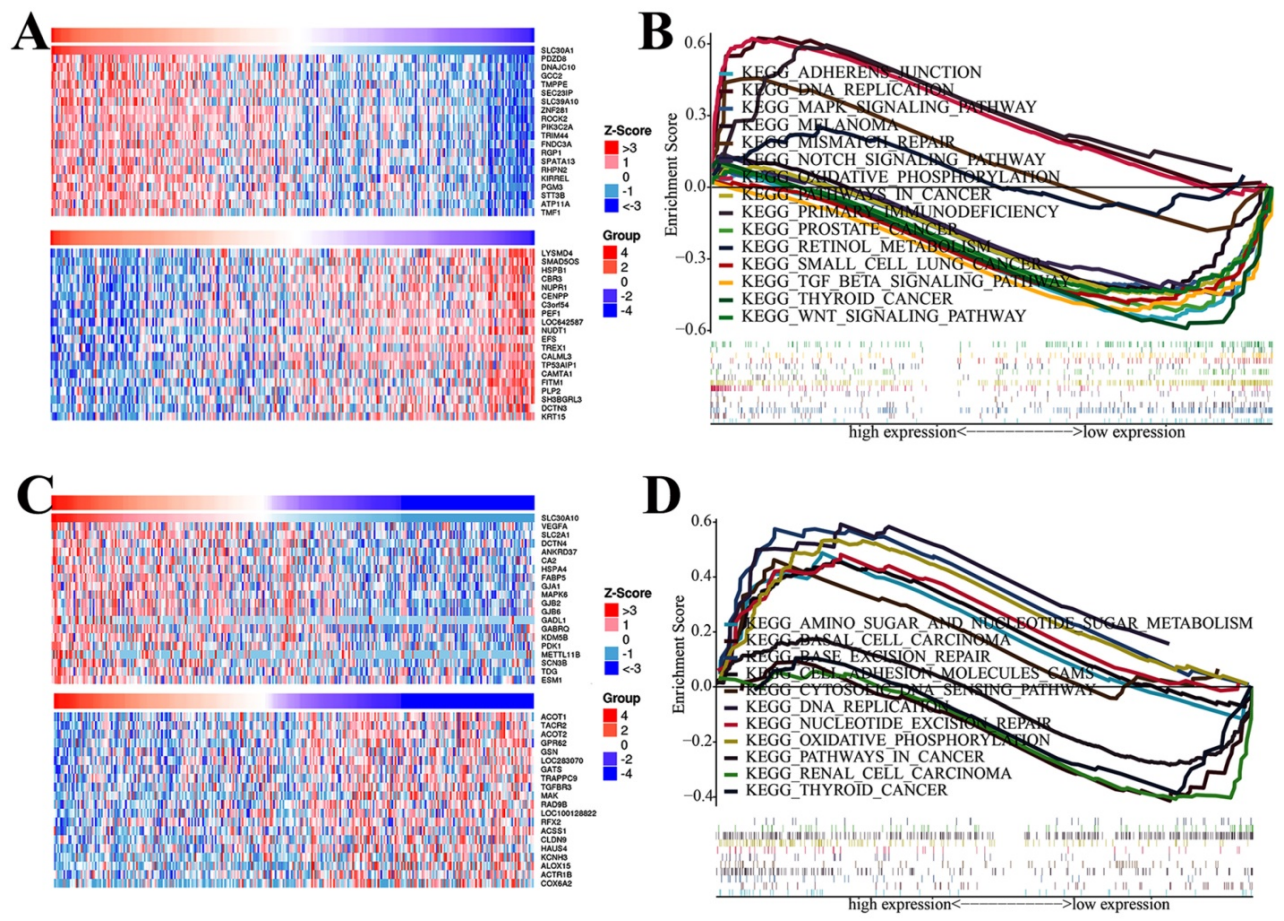
The top-20 correlated genes and GSEA analysis of SLC30A1/10 in cervical carcinoma. (A, C) The heatmap showing the top 20 genes positively or negatively correlated with SLC30A1 or SLC30A10. (B, D) GSEA analysis of SLC30A1 or SLC30A10.

To better elucidate the potential regulatory mechanisms of the SLC30A1 and SLC30A10 in cervical cancer, the LinkedOmics platform was used. The top-20 genes positively and negatively correlated with SLC30A1 and SLC30A 10 were shown in the Figure 9A and 9C, respectively. The top-20 genes positively associated with SLC30A1 were PDZD8, DNAJC10, GCC2, TMPPE, SEC23IP, SLC39A10, ZNF281, ROCK2, PIK3C2A, TRIM44, FNDC3A, RGP1, SPATA13, RHPN2, KIRREL, PGM3, STT3B, ATP11A, TMF1, and PPP4R2; the top-20 genes negatively associated with SLC30A1 were LYSMD4, SMAD5OS, HSPB1, CBR3, NUPR1, CENPP, C3orf54, PEF1, LOC642587, NUDT1, EFS, TREX1, CALML3, TP53AIP1, CAMTA1, FITM1, PLP2, SH3BGRL3, DCTN3 and KRT15. For SLC30A10, the positive genes were VEGFA, SLC2A1, DCTN4, ANKRD37, CA2, HSPA4, FABP5, GJA1, MAPK6, GJB2, GJB6, GADL1, GABRQ, KDM5B, PDK1, METTL11B, SCN3B, TDG, ESM1 and AGFG1; the negative genes were ACOT1, TACR2, ACOT2, GPR62, GSNL, OC283070, GATS, TRAPPC9, TGFBR3, MAK, RAD9B, LOC100128822, RFX2, ACSS1, CLDN9, HAUS4, KCNH3, ALOX15, ACTR1B and COX6A2.

As summarized in Table 4, for SLC30A1, the most correlated microRNA-targets were MIR-205, MIR-186, MIR-448, MIR-181A, MIR-302C, MIR-32, MIR-145, MIR-126, MIR-9, MIR-25, MIR-154, MIR-153, MIR-219, MIR-515-5P, MIR-133A, MIR-520D, MIR-101, MIR-369-3P, MIR-410, MIR-141, MIR-380-3P, MIR-26A, MIR-30A-5P, MIR-139, MIR-218, MIR-32, MIR-92, MIR-363, MIR-367, MIR-181B, MIR-181C, MIR-181D, MIR-487, MIR-519E, MIR-133B, MIR-200A, MIR-26B, MIR-30C, MIR-30D, MIR-30B and MIR-30E-5P; the most correlated transcript factor-targets were V$ATF6_01, V$HTF_01, V$HNF1_C, V$HTF_01 and V$HNF1_C. And the results showed that these transcript factors were primarily involved in the regulation of endocrine system development, positive regulation of nuclease activity, regulation of nuclease activity, cellular response to topologically incorrect protein, ER-nucleus signaling pathway, transcription initiation from RNA polymerase II promote (Figure S2). For SLC30A10, the most correlated microRNA-targets were MIR-200B, MIR-224, MIR-30A-3P, MIR-507, MIR-26A, MIR-518A-2, MIR-374, MIR-493, MIR-199A, MIR-448, MIR-488, MIR-380-3P, MIR-381, MIR-126, MIR-323, MIR-519E, MIR-202, MIR-362, MIR-194, MIR-155, MIR-182, MIR-527, MIR-186, MIR-101, MIR-369-3P, MIR-200C, MIR-429, MIR-30E-3P, MIR-26B and MIR-199B; the most correlated transcript factor-targets were V$HTF_01, V$ETF_Q6, V$E2F_Q2, V$MYOGNF1_01, TGASTMAGC_V$NFE2_01, V$AR_02, V$E2F_01, V$MAZ_Q6, V$IRF1_Q6, V$HMX1_01, V$AP1_Q6_01, V$ARNT_02, V$NKX61_01, GCCATNTTG_V$YY1_Q6, V$TCF11MAFG_01 and V$HNF6_Q6 (Table 5). The functions of these transcription factors were primarily participating in regulation region DNA binding, transcription coactivator activity, transcription regulatory region DNA binding (Figure S3).

To identify cancer-related signaling pathways associated with SLC30A1/10 in cervical cancer, we performed GSEA analysis. As shown in Figure 9B, the differential expression of SLC30A1 gene functions were mainly related to adherens junction, DNA replication, MAPK signaling pathway, melanoma, mismatch repair, Notch signaling pathway, oxidative, phosphorylation, pathways in cancer, primary immunodeficiency, prostate cancer, retinol metabolism, small cell lung cancer, TGF beta signaling pathway, thyroid cancer and WNT signaling pathway. And the differential expression functions of the SLC30A10 gene were primarily associated with amino sugar and nucleotide sugar metabolism, basal cell carcinoma, base excision repair, cell adhesion molecules cams, DNA replication, nucleotide excision repair, oxidative phosphorylation, pathways in cancer, renal cell carcinoma, and thyroid cancer. Besides, in order to further identify diseases caused by aberrant expression of the SLC30A1/10, the Open Targets website was used to perform disease analysis. Our results validated that the SLC30A1/10 was associated with the pathogenesis of immune diseases and many solid cancers (Figure S4-S5, Table S1-S2).

**Figure 10:**
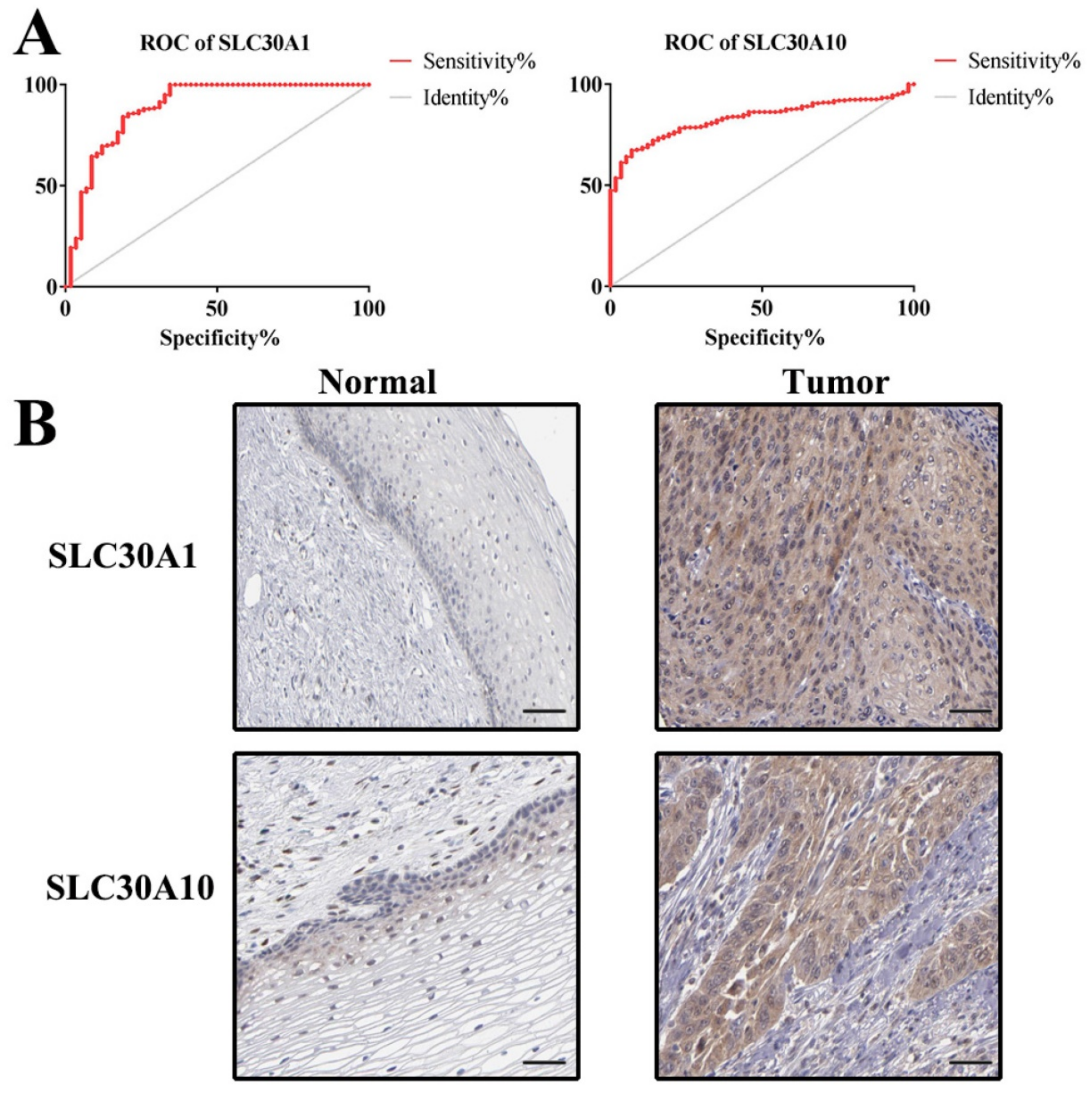
ROC curves and immunohistochemistry results of SLC30A1 and SLC30A10 in cervical carcinoma. (A) SLC30A1, AUC=0.8878 (95%CI: 0.8309 - 0.9448), p < 0.0001; SLC30A10, AUC=0.8314 (95% CI: 0.7939 - 0.8689), p <0.0001. (B) SLC30A1 and SLC30A10 proteins were higher in cervical carcinoma, compared to tumor-adjacent tissues.

**Figure 11:**
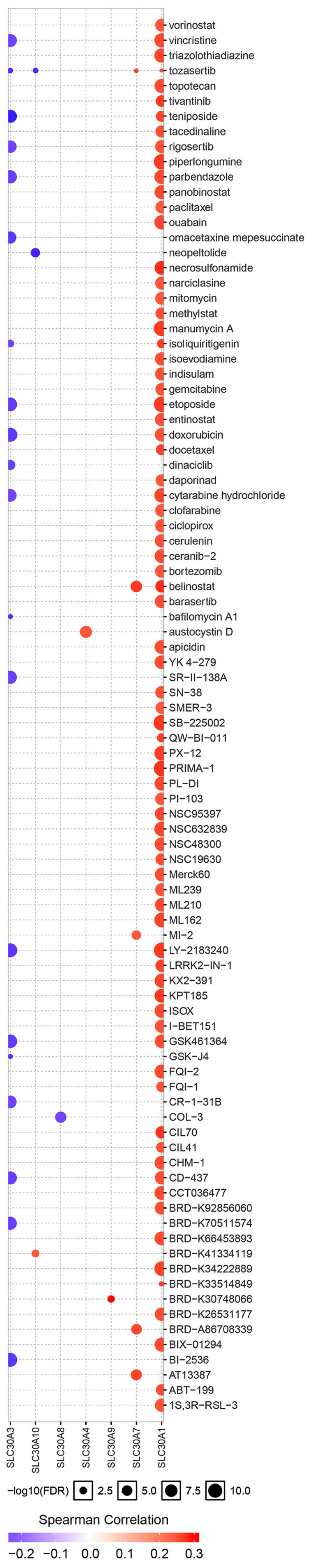
Drug susceptibility analysis of SLC30A1-10 genes. A positive correlation between gene expression and drug indicates that the gene has an antagonistic effect on the drug.

Additionally, ROC curves were performed to evaluate the diagnostic effects of SLC30A1 and SLC30A10 in cervical carcinoma. SLC30A1 and SLC30A10 can effectively distinguish cervical carcinoma patients, respectively (Figure 10A). The AUC of SLC30A1 was 0.8878 (95% CI: 0.8309 - 0.9448, p <0.0001) and the AUC of SLC30A10 was 0.8314 (95% CI: 0.7939 - 0.8689, p <0.0001). And we investigated the expression of SLC30A1 and SLC30A10 in cervical carcinoma by performing immunohistochemical staining on 31 pairs of tissues. The results showed that the expression of SLC30A1 and SLC30A10 was elevated in cervical carcinoma tissues compared with paracancerous tissues (Figure 10B). Finally, we evaluated the correlation between SLC30A1/10 expression and the IC50 of Cancer Drug Sensitivity Genomics (GDSC). In addition, the results showed that high expression of SLC30A1 was resistant to 79 drugs; two drugs (Neopeltolide and Tozasertib) can inhibit the high expression of SLC30A10 in cancers (Figure 11). Therefore, SLC30A1 and SLC30A10 can be used as potential diagnostic indicators and therapeutic targets in cervical carcinoma.

## Discussion

Skrajnowska et al indicated that zinc (Zn^2+^) was involved in the regulation of cancer immunoregulation and the metastasis of many cancers by modulating components of the tumor microenvironment 6. The SLC30 family genes, consisting of 10 members (SLC30A1-10), regulated zinc levels in human cells. Guo et al demonstrated that SLC30 family genes engaged in the regulation of the immune microenvironment in gastric cancer 8. In particular, SLC30A2 and SLC30A3 may play a pro-metastatic role directly 8. Moreover, Henshall et al showed that the expression of SLC30A4 was elevated in prostate cancer compared to paracancerous tissue and the expression of SLC30A4 in prostate cancer was negatively correlated with the immunoreactivity intensity 11. At present, there were some reports on the role of SLC30 family genes. However, the functions of SLC30 family genes in cervical carcinoma remained unclear. In other words, our study was the first one to investigate the roles of SLC30A1-10 genes in cervical carcinoma.

We first investigated the expression of the SLC30 family genes in cervical carcinoma. Data in the UALCAN tool revealed that mRNA expressions of SLC30A1, SLC30A7 and SLC30A10 were significantly higher in cervical cancer tissues, while SLC30A2 and SLC30A8 mRNA levels were decreased compared to normal tissues. For tumor stages, SLC30A1, SLC30A7 and SLC30A10 groups significantly varied. Besides, we found that SLC30A1, SLC30A7 and SLC30A10 expression was positively correlated with lymph node metastasis or distant metastasis in cervical carcinoma. Liu et al showed that SLC30A1 expression was significantly increased and the proliferative capacity of tumor cells was decreased after silencing the expression of SLC30A1 in bladder cancer 24. In addition, miRNA-411 can potentially modulate the malignant biological behavior of bladder cancer cells by targeting and regulating SLC30A1 expression 24. And the expression of SLC30A7 was also significantly elevated in hepatocellular carcinoma 25. These results indicated that SLC30 family genes were aberrantly expressed in a variety of cancers, suggesting that SLC30 family genes could have potential cancer regulatory roles.

To investigate the roles of the SLC30 family genes in cervical carcinoma, genomic changes and gene regulatory networks tightly linked to SLC30 family genes were analyzed. The results showed that SLC30 family genes were altered in 130 samples of 275 patients with cervical carcinoma (44%). Next, we performed functional enrichment analysis of these genes by using Funrich software and the Metascape platform. The results showed that the functions of these genes were concentrated on ion transport, regulation of translation, energy pathways, metabolism, regulation of nucleobase, cell growth and signal transduction. In addition, the SLC30 family genes can be potentially responsible for oncogenic or tumor suppressive regulation by participating in various cancer-related pathways. Gartmann et al demonstrated experimentally that aberrant expression of the SLC30A9 gene may exacerbate the growth and proliferative capacity of glioblastoma cells 26. It has been shown that many cancer cells can take up zinc from the blood or tumor microenvironment to play a role in promoting tumor formation and metastasis 27, 28. Besides, SLC30 family genes regulated zinc ion concentration in human cells. Thus, these findings all indicated that the SLC30 family genes may be associated with tumorigenesis.

However, after comprehensive integrative analyses of SLC30A1-10 genes in cervical carcinoma, we found that only two genes, SLC30A1 and SLC30A10, may potentially play a pro-cancerous role in cervical cancer. Next, we specifically explored the potential cancer-promoting mechanisms of these two genes.

There were increasing evidences that zinc ions were important for regulating the innate immune response, defending against invading pathogenic microorganisms and resisting the transformation from normal cells to cancer cells 29. Therefore, the correlation of SLC30A1 and SLC30A10 expression with immune cells and biomarkers in cervical cancer were investigated using the TISIDB platform. These results revealed that SLC30A1 and SLC30A10 were both associated with immune cells, immunostimulators, immunoinhibitors and MHC molecules, suggesting that SLC30A1/10 may contribute to the malignant behavior of cervical cancer cells by influencing immune factors. Consistent with our findings, Guo et al noted that SLC30A1 and SLC30A10 had prognostic values in gastric cancer 8. And SLC30A1 enhanced the metastatic ability and proliferative capacity of bladder cancer BiU87 cells by upregulating the expression of MMP-2 and cyclin D1 24. Up-regulation of SLC30A1 counteracted apoptotic effects of microRNA-8073 mimics on SKOV3 and OVCAR3 cell lines, suggesting that SLC30A1 has anti-apoptotic effects 9. Similarly, several reports suggested that SLC30A10 was a potential methylation biomarker for colorectal cancer 30-31. Besides, top-20 co-expression genes, kinases, transcription factors and miRNAs of SLC30A1 and SLC30A10 were analyzed using the LinkedOmics platform. In the transcription factor networks, we found that ATF6 was a key transcription factor that regulated SLC30A1; For SLC30A10, IRF1 and E2F1 were key transcription factors. Vahidi et al indicated that the expression of ATF6 fluctuated with the concentration of zinc ions 32. And it has been previously reported that IRF1 and E2F1 can regulate the proliferation and metastasis of various cancer cells by affecting their downstream genes 33-37. Furthermore, functions of these transcription factors were mainly enriched in regulatory RNA transcription, suggesting that SLC30A1/10 may have the ability to participate in the regulation of invasion and metastasis at the transcriptional level in cancers.

Then, in order to identify cancer-related signaling pathways associated with SLC30A1/10 genes in cervical cancer, we performed GSEA analysis according to SLC30A1/10 gene expression. The differential expression of SLC30A1 functional enrichments were mainly related to adherens junction, DNA replication, MAPK signaling pathway, mismatch repair, Notch signaling pathway, phosphorylation, pathways in cancer, primary immunodeficiency, prostate cancer, lung cancer, TGF beta signaling pathway, thyroid cancer and WNT signaling pathway. And the differential expression of SLC30A10 functions were primarily associated with basal cell carcinoma, base excision repair, cell adhesion molecules cams, cytosolic dna sensing pathway, DNA replication, nucleotide excision repair, oxidative phosphorylation, pathways in cancer, renal cell carcinoma and thyroid cancer. These results all demonstrated that the SLC30A1 and SLC30A10 had potential regulatory functions in cancers and were consistent with the results of previous literatures 38-41. And we validated the expression of SLC30A1 and SLC30A10 in cervical carcinoma by performing immunohistochemical staining on 31 pairs of tissues. The results showed that the expression of SLC30A1 and SLC30A10 was elevated in cervical carcinoma tissues compared with paracancerous tissues. Finally, we found that high expression of SLC30A1 was resistant to 79 drugs or small molecules. Two drugs (Neopeltolide and Tozasertib) can inhibit the high expression of SLC30A10 in cancers. Therefore, SLC30A1 and SLC30A10 can be used as potential diagnostic indicators and therapeutic targets in cervical carcinoma.

## Supplementary Material

Supplementary figures and tables.

## Figures and Tables

**Table 1:** The clinicopathological features of 31 patients.

Patient characteristics	No. of patients (%)
Age (years)	
<55	20(64.5)
≥55	11(35.5)
Pathological grade	
I + II	26(83.8)
III +IV	5(16.2)
Clinical stages	
Stage I and II	0(0)
Stage III	31(100)

**Table 2:** Correlations between expression of SLC30A1 with immune infiltration level in cervical cancer.

Gene	Infiltration	Analysis tools	r	P-value
SLC30A1	T cell CD4+ effector memory	XCELL	-0.169	0.005
SLC30A1	T cell CD4+ memory resting	CIBERSORT	0.219	0.000
SLC30A1	T cell CD4+ memory resting	CIBERSORT-ABS	0.238	0.000
SLC30A1	T cell CD4+ naive	XCELL	-0.250	0.000
SLC30A1	T cell CD4+ Th1	XCELL	-0.368	0.000
SLC30A1	T cell CD4+ Th2	XCELL	0.162	0.007
SLC30A1	T cell CD8+ central memory	XCELL	-0.164	0.006
SLC30A1	T cell CD8+ effector memory	XCELL	-0.313	0.000
SLC30A1	T cell CD8+ naive	XCELL	-0.200	0.001
SLC30A1	T cell CD8+	CIBERSORT	-0.144	0.017
SLC30A1	T cell CD8+	EPIC	0.187	0.002
SLC30A1	T cell CD8+	XCELL	-0.148	0.013
SLC30A1	B cell memory	CIBERSORT	-0.370	0.000
SLC30A1	B cell memory	CIBERSORT-ABS	-0.349	0.000
SLC30A1	B cell memory	XCELL	-0.253	0.000
SLC30A1	B cell naive	CIBERSORT	0.287	0.000
SLC30A1	B cell naive	CIBERSORT-ABS	0.282	0.000
SLC30A1	B cell naive	XCELL	-0.172	0.004
SLC30A1	B cell plasma	CIBERSORT	0.139	0.021
SLC30A1	B cell plasma	CIBERSORT-ABS	0.183	0.002
SLC30A1	B cell	XCELL	-0.326	0.000
SLC30A1	Class-switched memory B cell	XCELL	-0.247	0.000
SLC30A1	Neutrophil	CIBERSORT	0.176	0.003
SLC30A1	Neutrophil	CIBERSORT-ABS	0.188	0.002
SLC30A1	Neutrophil	MCPCOUNTER	0.362	0.000
SLC30A1	Monocyte	QUANTISEQ	-0.120	0.045
SLC30A1	Monocyte	XCELL	-0.243	0.000
SLC30A1	Macrophage M1	XCELL	-0.233	0.000
SLC30A1	Macrophage M2	CIBERSORT	-0.125	0.038
SLC30A1	Macrophage M2	XCELL	-0.200	0.001
SLC30A1	Macrophage	EPIC	-0.16502	0.005905
SLC30A1	Macrophage	XCELL	-0.21261	0.000366

**Table 3:** Correlations between expression of SLC30A10 with immune infiltration level in cervical cancer.

Gene	Infiltration	Analysis tools	r	P-value
SLC30A10	T cell CD8+	CIBERSORT-ABS	-0.136	0.023
SLC30A10	T cell regulatory (Tregs)	QUANTISEQ	-0.130	0.031
SLC30A10	B cell memory	XCELL	-0.192	0.001
SLC30A10	B cell	EPIC	-0.123	0.040
SLC30A10	B cell	MCPCOUNTER	-0.151	0.012
SLC30A10	B cell	XCELL	-0.165	0.006
SLC30A10	Class-switched memory B cell	XCELL	-0.120	0.045
SLC30A10	Neutrophil	MCPCOUNTER	0.135	0.025

**Table 4:** The Kinase, miRNA and transcription factor-target networks of SLC30A1 in cervical carcinoma (LinkedOmics).

Enriched Category	Gene Set	Size	Leading Edge Number	FDR
miRNA Target	ATGAAGG,MIR-205	147	66	0
	ATTCTTT,MIR-186	252	94	0
	ATATGCA,MIR-448	200	80	0
	TGAATGT,MIR-181A,MIR-181B,MIR-181C,MIR-181D	449	191	0
	ATGTTAA,MIR-302C	228	72	0
	TAATGTG,MIR-323	148	60	0
	AACTGGA,MIR-145	215	75	0
	TAATAAT,MIR-126	207	83	0
	TAGCTTT,MIR-9	224	80	0
	GTGCAAT,MIR-25,MIR-32,MIR-92,MIR-363,MIR-367	294	90	0
	GTATGAT,MIR-154,MIR-487	67	29	0
	CTATGCA,MIR-153	199	58	0
	GACAATC,MIR-219	134	57	0
	TTGGAGA,MIR-515-5P,MIR-519E	130	50	0
	GGGACCA,MIR-133A,MIR-133B	183	74	0
	TTTGTAG,MIR-520D	320	105	9.22E-05
	GTACTGT,MIR-101	236	84	9.56E-05
	GTATTAT,MIR-369-3P	193	71	9.93E-05
	GTTATAT,MIR-410	85	35	0.000103
	CAGTGTT,MIR-141,MIR-200A	292	105	0.000108
	ATTACAT,MIR-380-3P	95	41	0.000112
	TACTTGA,MIR-26A,MIR-26B	285	102	0.000117
	TGTTTAC,MIR-30A-5P,MIR-30C,MIR-30D,MIR-30B,MIR-30E-5P	545	181	0.000123
	ACTGTAG,MIR-139	116	44	0.000129
	AAGCACA,MIR-218	375	139	0.000136
Transcription Factor Target	V$ATF6_01	117	38	0.004472
	V$HTF_01	65	21	0.019008
	V$HNF1_C	222	61	0.020126

**Table 5:** The Kinase, miRNA and transcription factor-target networks of SLC30A10 in cervical carcinoma (LinkedOmics).

Enriched Category	Gene Set	Size	Leading Edge Number	FDR
miRNA Target	CAGTATT,MIR-200B,MIR-200C,MIR-429	442	154	0
	GTGACTT,MIR-224	150	67	0
	ACTGAAA,MIR-30A-3P,MIR-30E-3P	185	62	0
	GTGCAAA,MIR-507	118	44	0
	TACTTGA,MIR-26A,MIR-26B	285	95	0.000586
	TTTGCAG,MIR-518A-2	193	56	0.000703
	TATTATA,MIR-374	267	95	0.000879
	ATGTACA,MIR-493	301	113	0.001005
	ACACTGG,MIR-199A,MIR-199B	146	45	0.001538
	ATATGCA,MIR-448	200	94	0.001641
	TATCTGG,MIR-488	56	26	0.001758
	ATTACAT,MIR-380-3P	95	39	0.001862
	CTTGTAT,MIR-381	187	67	0.001893
	TAATAAT,MIR-126	207	72	0.002051
	TAATGTG,MIR-323	148	55	0.00211
	GGCACTT,MIR-519E	113	42	0.002238
	ATAGGAA,MIR-202	94	36	0.002344
	CAAGGAT,MIR-362	63	23	0.002344
	CTGTTAC,MIR-194	97	41	0.002446
	AGCATTA,MIR-155	128	36	0.002546
	TTGCCAA,MIR-182	299	76	0.002557
	CTTTGCA,MIR-527	222	70	0.00257
	ATTCTTT,MIR-186	252	96	0.002579
	GTACTGT,MIR-101	236	75	0.002605
	GTATTAT,MIR-369-3P	193	66	0.002637
Transcription Factor Target	V$HTF_01	65	28	0.000512
	KCCGNSWTTT_UNKNOWN	95	33	0.000614
	V$ETF_Q6	105	43	0.000768
	V$E2F_Q2	161	65	0.001024
	V$MYOGNF1_01	43	13	0.001536
	SMTTTTGT_UNKNOWN	368	96	0.003072
	KRCTCNNNNMANAGC_UNKNOWN	62	33	0.004306
	TGASTMAGC_V$NFE2_01	183	51	0.005267
	V$AR_02	33	7	0.007681
	V$E2F_01	64	22	0.017941
	V$MAZ_Q6	180	52	0.019457
	V$IRF1_Q6	237	66	0.019585
	V$HMX1_01	39	21	0.019903
	GGCKCATGS_UNKNOWN	61	19	0.019969
	V$AP1_Q6_01	252	57	0.020212
	V$ARNT_02	228	58	0.02024
	V$NKX61_01	217	53	0.020768
	GCCATNTTG_V$YY1_Q6	387	95	0.020774
	TMTCGCGANR_UNKNOWN	149	38	0.021506
	ATGGYGGA_UNKNOWN	94	31	0.021659
	V$TCF11MAFG_01	193	61	0.02253
	SYATTGTG_UNKNOWN	211	54	0.022734
	V$HNF6_Q6	219	55	0.024203
