# Supplementary material for: The correlation and role analysis of SLC30A1 and SLC30A10 in cervical carcinoma
Source: J Cancer. 2022 Jan 4;13(3):1031–47. doi: 10.7150/jca.56777 (PMC8824890; doi:10.7150/jca.56777)
Supplement: Supplementary file 1 — Supplementary figures and tables. [file jcav13p1031s1.pdf]

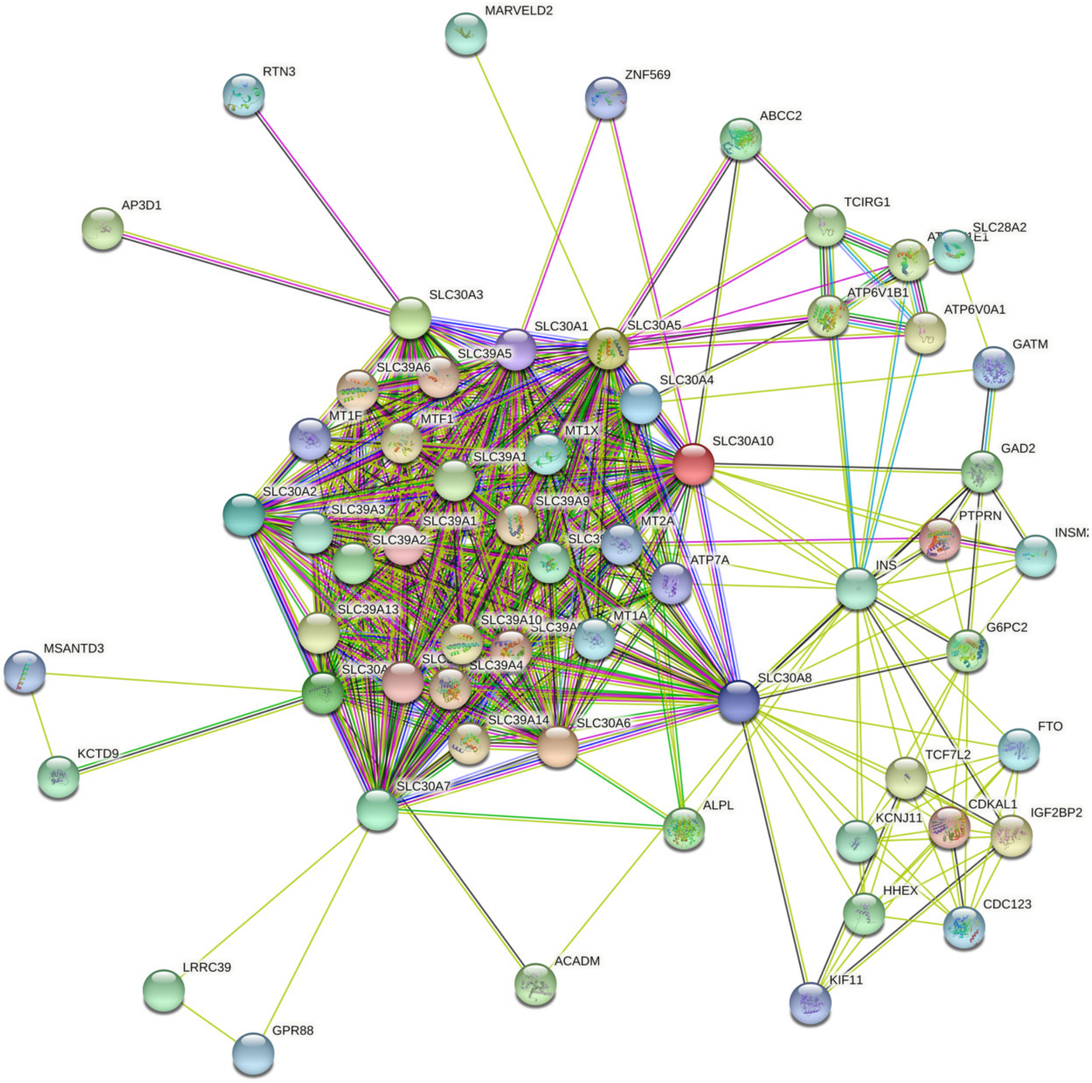

Figure S1. Neighbor gene network of SLC30 family genes in cervical squamous cell carcinoma.

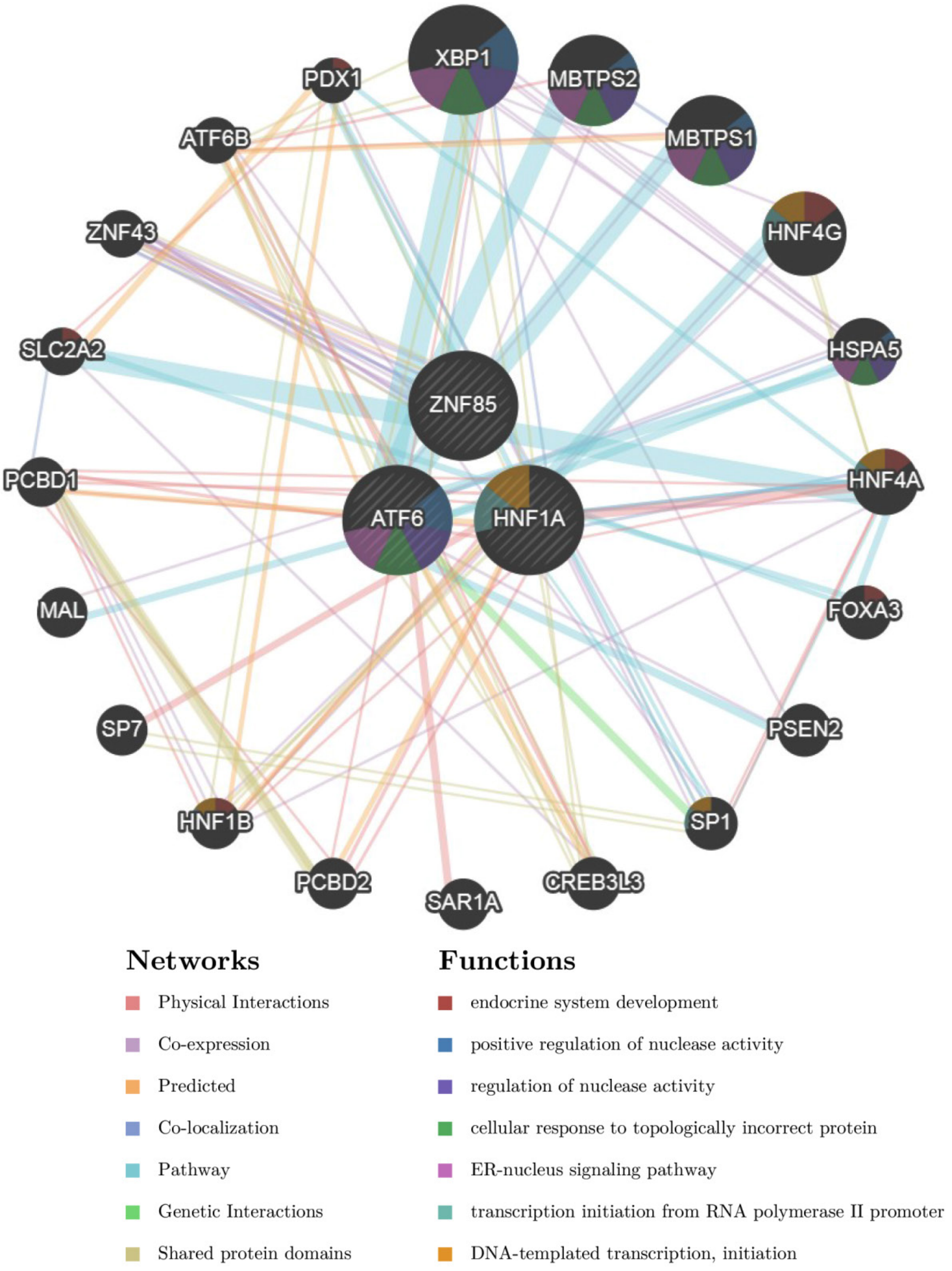

Figure S2. PPI network of transcription factors of SLC30A1 target networks (GeneMANIA).

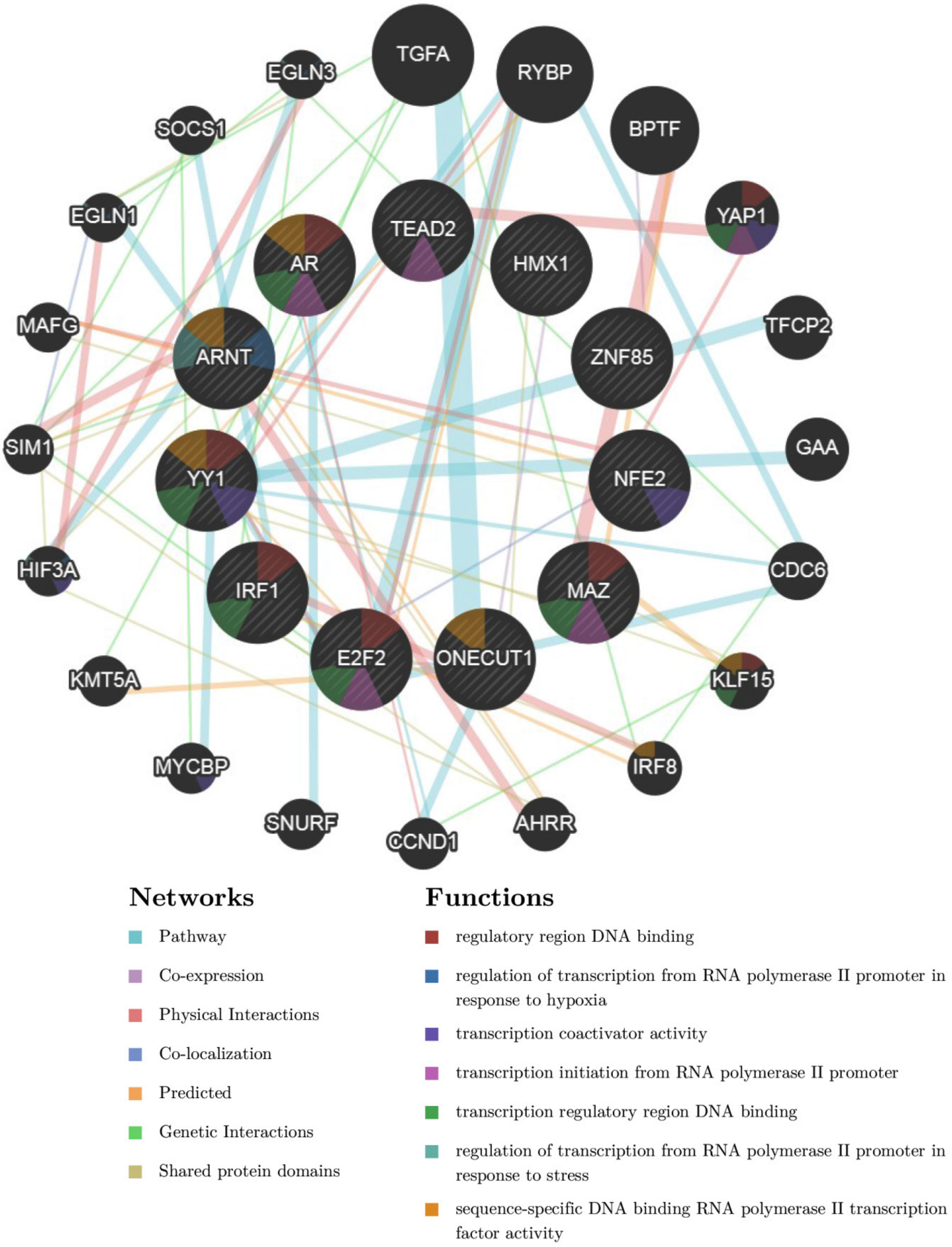

Figure S3. PPI network of transcription factors of SLC30A10 target

networks (GeneMANIA).

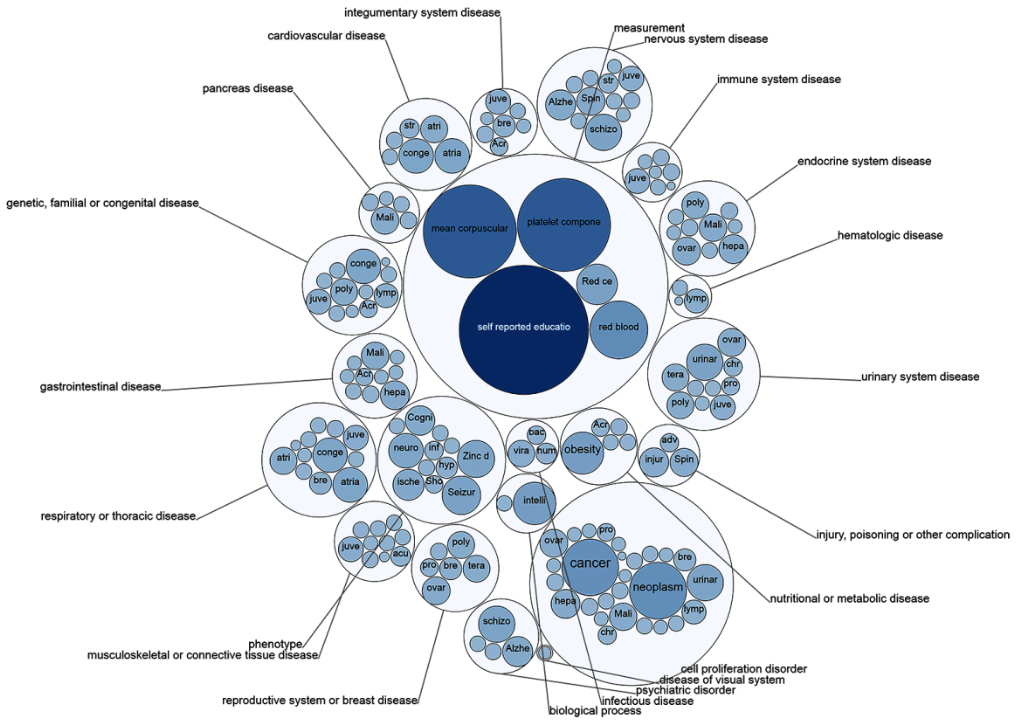

Figure S4. The diseases related to SLC30A1 (Open Targets).

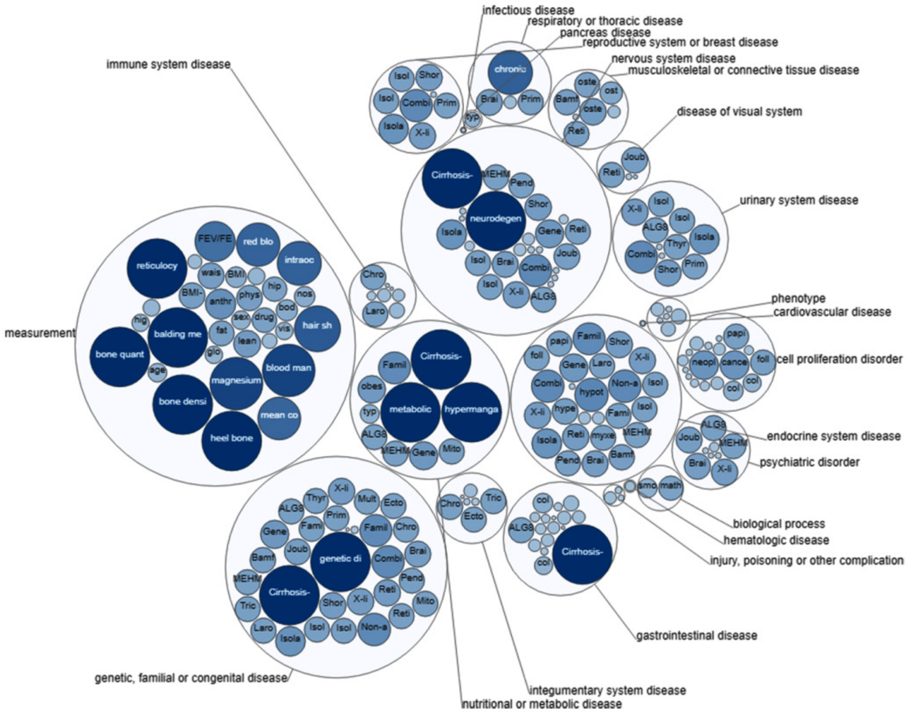

Figure S5. The diseases related to SLC30A10 (Open Targets).

Table S1. The diseases related to SLC30A1 (Open Targets).

Disease label	Association score
neoplasm	0.1409889
cancer	0.1396609
urinary bladder cancer	0.0572
hepatocellular carcinoma	0.0363021
ovarian carcinoma	0.0341
Malignant Pancreatic Neoplasm	0.0329
lymphoma	0.0252
breast cancer	0.021375
chromophobe renal cell carcinoma	0.0146304
adverse effect	0.0145
prostate adenocarcinoma	0.0137
pancreatic ductal adenocarcinoma	0.0122222
invasive breast ductal carcinoma	0.0121955
lung cancer	0.0112
glioma	0.0105
colon adenocarcinoma	0.0084
pancreatic adenocarcinoma	0.0084
rectal adenocarcinoma	0.0084
esophageal carcinoma	0.0084
thyroid carcinoma	0.0084
glioblastoma multiforme	0.0084
gastric adenocarcinoma	0.0084

Table S2. The diseases related to SLC30A10 (Open Targets).

Disease label	Association score
neoplasm	0.240721
cancer	0.238301
papillary thyroid carcinoma	0.18074
follicular thyroid carcinoma	0.18074
colorectal cancer	0.110771
colorectal adenocarcinoma	0.093704
colorectal neoplasm	0.052206
colorectal carcinoma	0.047178
colonic neoplasm	0.027243

neuroblastoma	0.024689
gastric carcinoma	0.020449
glioma	0.013
grade II glioma	0.0104
adrenal cortex carcinoma	0.010306
uterine leiomyosarcoma	0.009839
